# Asymmetric Dimethylarginine Is Associated with Developmental Programming of Adult Kidney Disease and Hypertension in Offspring of Streptozotocin-Treated Mothers

**DOI:** 10.1371/journal.pone.0055420

**Published:** 2013-02-07

**Authors:** You-Lin Tain, Wen-Chin Lee, Chien-Ning Hsu, Wei-Chia Lee, Li-Tung Huang, Chien-Te Lee, Ching-Yuang Lin

**Affiliations:** 1 Departments of Pediatrics, Kaohsiung Chang Gung Memorial Hospital, Chang Gung University College of Medicine, Kaohsiung, Taiwan; 2 Nephrology, Kaohsiung Chang Gung Memorial Hospital, Chang Gung University College of Medicine, Kaohsiung, Taiwan; 3 Pharmacy, Kaohsiung Chang Gung Memorial Hospital, Chang Gung University College of Medicine, Kaohsiung, Taiwan; 4 Graduate Institute of Clinical Pharmacy, College of Pharmacy, Kaohsiung Medical University, Kaohsiung, Taiwan; 5 Devision of Urology, Kaohsiung Chang Gung Memorial Hospital, Chang Gung University College of Medicine, Kaohsiung, Taiwan; 6 College of Medicine, China Medical University, Clinical Immunological Center, and Division of Pediatric Nephrology, China Medical University Hospital, Taichung, Taiwan; National Cancer Institute, United States of America

## Abstract

Diabetes mellitus complicates pregnancies, leading to diseases in adult life in the offspring. Asymmetric dimethylarginine (ADMA) is increased in diabetes mellitus, kidney disease, and hypertension. We tested whether maternal diabetes causes increased ADMA in rats, resulting in kidney disease and hypertension in the adult offspring, and whether these can be prevented by maternal citrulline supplementation. Newborn female and pregnant Sprague-Dawley rats were injected with streptozotocin (STZ), which made up the nSTZ and STZ models, respectively. For the STZ model, 4 groups of male offspring were killed at age 3 months: the control, STZ, and Cit and STZ+Cit (control and STZ rats treated with 0.25% l-citrulline solution, respectively) groups. The nSTZ rats had lower nephron numbers. The renal level of ADMA was higher in the nSTZ rats than in controls. The STZ group developed kidney injury, renal hypertrophy, and elevated blood pressure at the age of 12 weeks. These conditions were found to be associated with increased ADMA levels, decreased nitric oxide (NO) production, and decreased dimethylarginine dimethylaminohydrolase (DDAH) activity in the kidney. In addition, ADMA caused a nephron deficit in cultured rat metanephroi. Maternal citrulline supplementation prevented hypertension and kidney injury, increased the renal DDAH-2 protein level, and restored the levels of ADMA and NO in the STZ+Cit group. Reduced nephron number and increased ADMA contribute to adult kidney disease and hypertension in offspring of mothers with STZ-induced diabetes. Manipulation of the ADMA-NO pathway by citrulline supplementation may be a potential approach to prevent these conditions.

## Introduction

The incidence of diabetes is increasing worldwide, including type 2 diabetes in women of reproductive age. Diabetes mellitus complicates pregnancies and is associated with high rates of impaired organogenesis and disease in the offspring later in life [1]. In addition, diabetes is one of the most common causes of chronic kidney disease (CKD). Considering the globally increasing incidence of both CKD and diabetes, both of which may originate early in life [2], a better understanding of the mechanisms of the developmental programming of adult kidney disease by exposure to maternal diabetes may reveal ideal targets for early prevention.

Evidence from human and animal studies suggests that maternal malnutrition causes developmental programming of adult kidney disease in the offspring [3]–[8]. This is mainly due to reduced nephron numbers and glomerular hypertrophy, which are caused by the imbalance between the nitric oxide (NO) and reactive oxygen species required for nephrogenesis [3], [4]. Asymmetric dimethylarginine (ADMA), an endogenous inhibitor of NO synthase (NOS), is involved in the development of CKD [9]. We found that 50% maternal caloric restriction causes low nephron numbers and renal dysfunction, which are associated with increased plasma ADMA level in the adult offspring [8]. In addition, elevated concentrations of ADMA have been reported in patients with different types of diabetes [10]–[13] and their related complications [14], [15]. However, little attention has been given on whether maternal diabetes impairs the ADMA-NO pathway, resulting in developmental programming in the kidneys of the offspring. Additionally, we found that supplementation with maternal citrulline (the precursor of arginine) prevented the development of adult kidney disease in a maternal caloric restriction model [8]. Because citrulline is mainly taken up by the kidney and metabolized into arginine, it is possible that citrulline supplementation increases arginine and competes with ADMA, thereby preventing NO deficiency [16]. Therefore, we hypothesized that maternal diabetes causes increased ADMA and NO deficiency, resulting in kidney disease in the adult offspring that can be prevented by maternal citrulline supplementation.

Streptozotocin (STZ)-induced diabetes in pregnant rodents reduces nephron numbers in the offspring, and induces hypertension and renal dysfunction in adulthood [6], [7], [17]. Unlike a single dose of STZ that can induce type 1 diabetes in adult rats, STZ injected neonatally (nSTZ) in rats induces type 2 diabetes in the adult age [18]. Therefore, we intended to elucidate whether ADMA is a major target in the developmental programming of adult kidney disease in offspring of diabetic mothers, by using the STZ- and nSTZ-induced diabetes rat models. We also intended to examine the effect of maternal citrulline supplementation on the ADMA-NO pathway in the kidneys of offspring exposed to maternal diabetes.

## Materials and Methods

### Animal Protocol

This study was approved and performed under the Guidelines for Animal Experiments of Chang Gung Memorial Hospital and Chang Gung University. The treatment of animals conformed to the Guide for the Care and Use of Laboratory Animals published by the U.S. National Institutes of Health. Virgin Sprague-Dawley (SD) rats (12–16 weeks old) were obtained (BioLASCO Taiwan Co., Ltd., Taipei, Taiwan), and then housed and maintained in a facility accredited by the Association for Assessment and Accreditation of Laboratory Animal Care International. Male SD rats were caged with individual females until mating was confirmed.

For the nSTZ model, newborn female SD rats (n = 10) received a single i.p. injection of 50 mg (per kilogram of rat body weight) STZ (Sigma, Steinheim, Germany) freshly dissolved in citrate buffer (0.05 mM, pH 4.5). The control group (n = 10) consisted of female offspring with only i.p. injection of citrate buffer. Pups were left with their mothers. All neonates were tested on day 2 for glycosuria. Only animals that were glycosuric at day 2 after birth were included in the nSTZ group. Rats were maintained under standard conditions with free access to tap water and standard rat chow for growing the animals until mating. Male SD rats were caged with individual females until mating was confirmed. Pregnant rats were maintained until delivery. Male offspring were killed at age 1 week. Kidneys were removed and flash frozen until analysis. Nephron number was counted using our previously published method [8].

For the STZ model, pregnant SD rats were made diabetic on day 0 of gestation by a single i.p. injection of 45 mg STZ (freshly dissolved in citrate buffer) per kilogram of body weight. Control rats were given an equivalent amount of citrate buffer. The diabetic state was confirmed by measuring the plasma glucose concentration 3 days after STZ injection. Only pregnant rats whose plasma glucose was >15 mmol/L were included. The STZ group consisted of 10 male offspring of 2 STZ-induced diabetic mothers. Another group of STZ offspring (STZ+Cit, n = 10) was prepared by treating STZ-induced diabetic mothers with 0.25% citrulline (Sigma, St. Louis, MO, USA) solution dissolved in drinking water during the whole period of pregnancy and lactation. The control group (n = 10) consisted of male offspring of control mothers with free access to standard rat chow. Another group of control offspring (Cit, n = 10) were set by treating control mothers with 0.25% citrulline in drinking water during pregnancy and lactation, as we have previously described [8].

All male offspring were weighed and evaluated for BP, and their urine was collected on postnatal weeks 4, 8, and 12. Systolic and diastolic BP were determined in conscious rats by an indirect tail-cuff method (BP-2000; Visitech Systems Inc., Apex, NC, USA) [8]. Rats were systematically trained and placed in restrainers several times before the measurements. Three stable consecutive measurements were taken and averaged. Twenty-four-hour urine collections were performed before sacrifice for the determination of total protein by the Bradford method and nitrite levels by Greiss reaction, as we have published previously [8]. Adult offspring were killed at age 12 weeks. Heparinized blood samples were collected at sacrifice. Kidney was harvested after perfusion with PBS. One kidney was removed, decapsulated, divided into the cortex and medulla, and snap frozen for western blot analysis; the other kidney was perfusion-fixed for histological analysis. Plasma creatinine levels were analyzed with high-performance liquid chromatography (HPLC). Twenty-four-hour urine collections were performed before sacrifice for the determination of total protein by Bradford method and total urine NO production (from NOx = NO_2_^−^ +NO_3_^−^ ) was measured by the Griess reaction as previously described [8].

### Histology and Morphometric Study

Four-micrometer sections of formalin-fixed, paraffin-embedded kidney were stained with periodic acid-Schiff. The extent of renal injury was assessed on a blinded basis by evaluating the glomerular injuries. Glomerular injury scores, representing sclerotic damage to glomeruli (n = 100), were calculated using the 0 to 4+ scale.

Tubulointerstitial injury scores were based on the presence of tubular cellularity, basement membrane thickening, dilation, atrophy, sloughing, or interstitial widening. Tubulointerstitial injury scores were graded as follows: 0, no changes; grade 1, <10% involvement; grade 2, 10–25% involvement; grade 3, 25–50% involvement; grade 4, 50–75% involvement; and grade 5, 75–100% involvement, as we previously published [8].

### Metanephros Organ Culture

Metanephros organ culture was performed as we previously described [19]. Briefly, SD female rats of known mating date were anesthetized and laparotomized. Fetuses were aseptically removed, and metanephroi from fetuses of embryonic day 14 (E14) were collected and freed of exogenous tissue. Explants were placed onto a Steritop filter unit (Millipore, Billerica, MA, USA), floating on a defined serum-free medium and incubated for 6 days in 35-mm Petri dishes at 37°C in a humidified incubator (5% CO_2_). The defined medium was composed of Eagle’s minimum essential medium with 10% (v/v) fetal calf serum, 100 units/mL penicillin, and 100 µg/mL streptomycin. All the above chemicals were from Sigma. The culture medium was changed daily, and no antibiotic or fungicide was present throughout the experiment. New aliquots of each additive were used at every new metanephros culture.

Metanephroi were treated with different concentrations of ADMA (2 and 10 µM) and harvested after 6 days for nephron counting (n = 6/group). The medium was changed daily. The metanephroi were carefully detached from the filter and rinsed sequentially in PBS. The explants were labeled with fluorescein-coupled helix pomatia agglutinin (1∶100; Invitrogen Taiwan Ltd., Taipei, Taiwan) and peanut agglutinin (1∶100, Invitrogen Taiwan Ltd.) for 1 h. After washing, the metanephric explants were mounted in PBS/glycerol. The total numbers of glomeruli were counted by 2 observers.

### HPLC Detection of Arginine, Citrulline, ADMA, and SDMA

The levels of arginine, citrulline, ADMA, and symmetric dimethylarginine (SDMA, a stereoisomer of ADMA) were measured using HPLC (HP series 1100; Agilent Technologies Inc., Santa Clara, CA, USA) with the OPA-3MPA derivatization reagent as we have previously described [20]. Homoarginine (Sigma) was used as the internal standard. The standards contained arginine, citrulline, ADMA, and SDMA at concentrations in the range of 1–100, 1–100, 0.5–5, and 0.5–5 µM, respectively. The recovery rate was approximately 90–105%. The tissue concentration was factored for protein concentration, which was represented as micromolars per milligram of protein.

### Western Blot

Western blot analysis was done as we have previously described [20]. A list of antibodies used for Western blotting was shown in Table S1. Bands of interest were visualized using ECL reagents (PerkinElmer, Waltham, MA, USA) and quantified by densitometry (Quantity One Analysis software; Bio-Rad), as integrated optical density (IOD) after subtraction of background. The IOD was factored for Ponceau red staining to correct for any variations in total protein loading. The protein abundance was represented as IOD/PonS.

### DDAH Activity

DDAH activity was measured by colorimetric assay, which determines the rate of citrulline production, as optimized by us recently [21]. Tissue sample was homogenized in sodium phosphate buffer. The homogenate was preincubated with urease for 15 min, and 100 µL (2 mg) of the homogenate was incubated with 1 mM ADMA for 45 min at 37°C. After deproteinization, the supernatant was incubated with color mixture at 60°C for 110 min. The absorbance was measured by spectrophotometry at 466 nm. DDAH activity was expressed as micromolars of citrulline generated per gram of protein per minute at 37°C.

### Detection of NO by EPR

NO was detected by electronic paramagnetic resonance (EPR) with spin trapping. Samples from tissues were prepared as described previously [22]. 10 µg of protein was added to 100 mM N-methyl-D-glucamine dithiocarbamate (MGD), 10 mM FeSO4, and 100 mM sodium dithionite in a total volume of 100 µl of Chelex-treated phosphate-buffered saline. In duplicate samples, MGD and FeSO4 were omitted. Samples were placed in 50 µl glass capillaries (Wilmad Glass, Buena, NJ). The ESR spectra was recorded using an EMX Plus EPR spectrometer (Bruker, Biospin, Rheinstetten, Germany) equipped with an EMX-m40X microwave bridge operating at 3.16 G.

### Statistics

Results were expressed as mean ± SEM. Morphological and biochemical parameters were analyzed by one-way ANOVA with post hoc least significant difference test. All analyses were performed using SPSS. A p value of <0.05 was considered statistically significant.

## Results

### nSTZ Model

**Figure 1:**
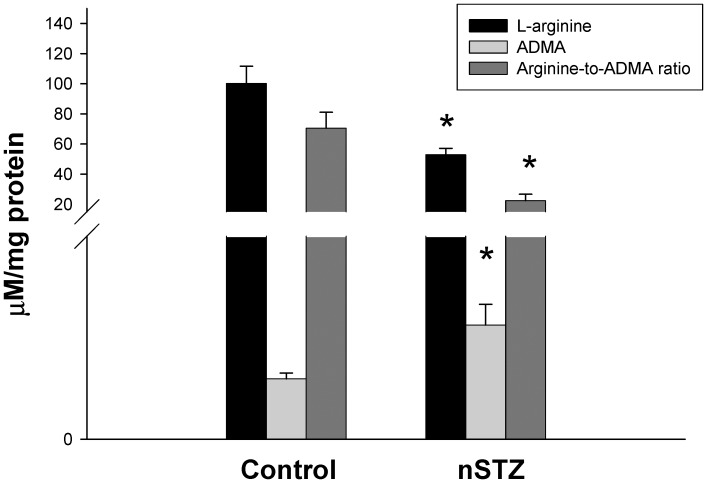
Alterations of renal arginine, ADMA, and arginine-to-ADMA ratio in the nSTZ offspring. Arginine, ADMA, and arginine-to-ADMA ratio in the kidneys of 1-week-old male offspring from nSTZ and control mothers. *p<0.05 vs. control.

**Figure 2:**
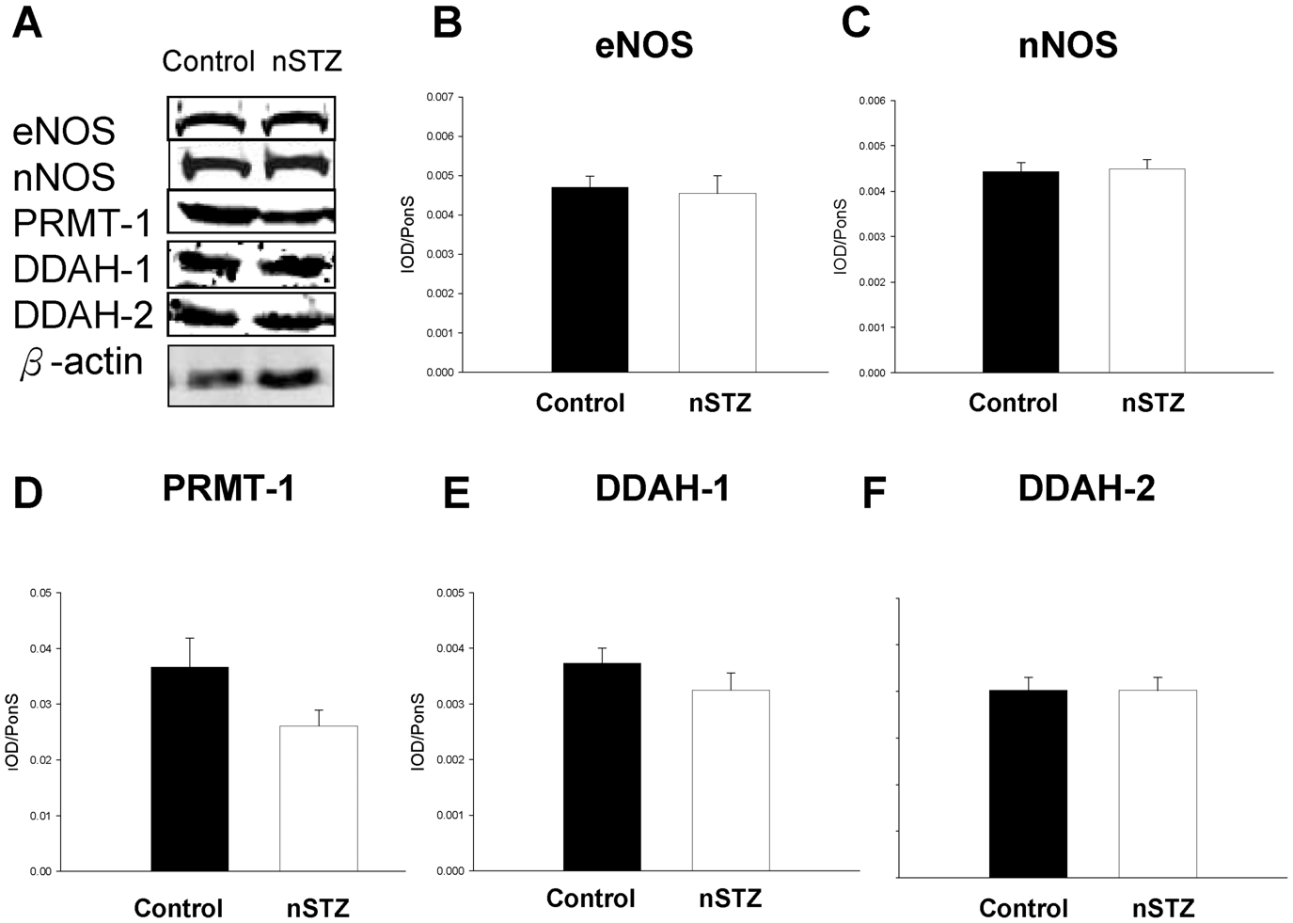
Protein levels of NOS and ADMA-related enzymes in the nSTZ offspring kidney. (A) Representative western blots showing eNOS (∼150 kDa), nNOS (∼160 kDA), PRMT-1 (∼42 kDa), DDAH-1 (∼34 kDa), and DDAH-2 (∼30 kDa) bands in control and offspring from nSTZ mothers at 1 week of age. The relative abundances of renal cortical (B) eNOS, (C) nNOS, (D) PRMT-1, (E) DDAH-1, and (F) DDAH-2 were quantified. n = 6/group.

The duration of gestation, number of newborns per litter, body weight (15.2±0.2 vs. 15.3±0.1 g, p = 0.96), and kidney weight (116±4 vs. 120±3 mg, p = 0.46) of the nSTZ rats did not differ from those of controls; however, the nephron number was 28% lower than that in controls at 1 week of age (141,550±17,689 vs. 197,500±19,031, p = 0.024). As shown in Figure 1, the renal ADMA level was elevated nearly 2-fold in 1-week-old nSTZ offspring; however, the arginine level and the arginine-to-ADMA ratio in the kidney were significantly decreased. In contrast, the protein levels of endothelial NOS (eNOS), neuronal NOS (nNOS), protein arginine methyltransferase (PRMT)-1 (ADMA-synthesizing enzyme), and dimethylarginine dimethyaminohydrolase (DDAH)-1 and -2 (ADMA-metabolizing enzymes) were not different between the nSTZ offspring and controls (Fig. 2). Renal DDAH activity was not analyzed in this model because of limited samples.

### STZ Model

Litter sizes were not significantly altered by STZ treatment of the maternal rat (pups per litter: control group = 13; STZ group = 12) or by citrulline supplementation (Cit group = 13.5; STZ+Cit group = 12.5). The amount of water intake and urine output were not significantly different between the control and Cit groups. The mortality rate of male pups was not different among the 4 groups.

**Figure 3:**
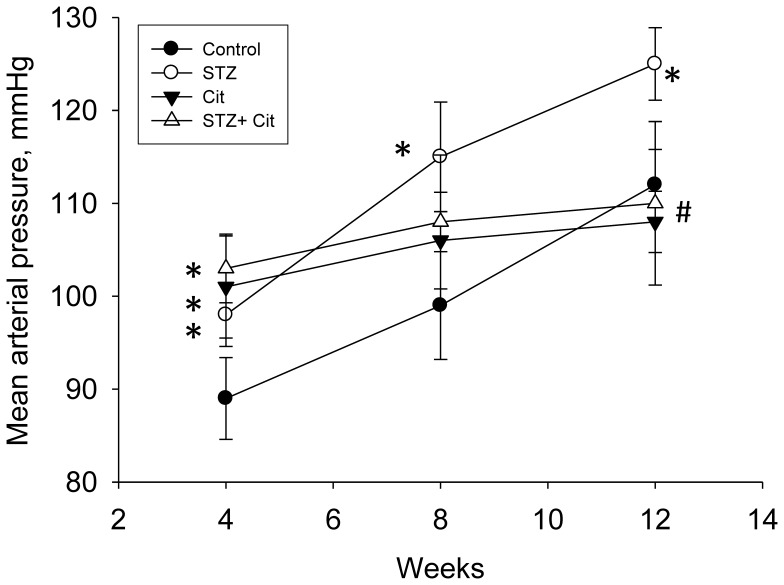
Effect of maternal citrulline supplementation on mean arterial blood pressure. Data are for controls and offspring from mothers treated with STZ, at different ages. STZ, offspring of STZ-induced diabetic mother; Cit, offspring of control mothers treated with 0.25% l-citrulline; STZ+Cit, offspring of STZ-induced diabetic mothers treated with 0.25% l-citrulline. *p<0.05 vs. control; #p<0.05 vs. STZ.

As shown in Table 1, the Cit group had higher body weights compared with the other 3 groups at 12 weeks of age. STZ treatment plus citrulline supplementation caused increased kidney weights and kidney weight-to-body weight ratios. However, there was no synergistic effect. Surprisingly, the mean arterial blood pressure (BP) was elevated in the STZ, Cit, and STZ+Cit groups compared with the control group at 4 weeks of age. However, the increase in mean arterial BP during the development of the STZ group from age 4 to 12 weeks was prevented by l-citrulline therapy (Fig. 3). At 12 weeks, the STZ offspring developed higher systolic and diastolic BP than controls; however, citrulline supplementation attenuated the elevation of diastolic but not systolic BP (Table 1). Proteinuria was not found in either group. Similarly, plasma creatinine level was not different among the 4 groups. These data demonstrated that STZ and Cit both induced renal hypertrophy and increased systolic BP, and had no effect on renal function.

**Table 1:** Weights and renal outcome parameters in STZ-induced diabetic offspring at 12 weeks of age.

	Control	STZ	Cit	STZ+Cit
	n = 10	n = 10	n = 10	n = 10
Body weight (g)	470±8.8	501.6±18.9	505.2±7^a^	484.5±11.9
Left kidney weight (g)	2±0.06	2.32±0.13^a^	2.26±0.04^a^	2.36±0.07^a^
Left KW/100 g BW	9.4±0.38	11.8±1.03^a^	11.4±0.88^a^	11.4±0.45^a^
Systolic BP (mm Hg)	114±2	142±4^a^	132±5^a^	135±10^a^
Diastolic BP (mm Hg)	93±6	112±5^a^	93±4	97±9
Urine protein excretion (mg⋅24 h^−1^⋅100 g^−1^ BW)	3.24±0.26	2.72±0.39	3.35±0.31	3.42±0.2
Plasma Cr (mg/dL)	0.28±0.02	0.29±0.04	0.32±0.04	0.27±0.02
Glomerular injury index	2.6±0.7	2.7±1.4	2.4±0.5	2.7±0.8
Tubulointerstitial injury index	0.45±0.04	1.65±0.15^a^	0.49±0.08^b^	0.59±0.18^b^

STZ, offspring of STZ-induced diabetic mother; Cit, offspring of control mother treated with 0.25% l-citrulline; STZ+Cit, offspring of STZ-induced diabetic mother treated with 0.25% l-citrulline. KW, kidney weight; BW, body weight; BP, blood pressure; Cr, creatinine.

a p<0.05 vs. control;

b p<0.05 vs. STZ.

The glomerular injury score was not significantly different among the groups but tended to be higher in the STZ group. Unlike the glomerular injury score, the tubulointerstitial injury score was higher in the STZ group compared with the other groups, showing that maternal citrulline supplementation prevented the increase of tubulointerstitial injury scores in the STZ group. STZ resulted in significant reductions in nephron number, whereas maternal l-citrulline therapy led to increased nephron number in both the Cit and STZ+Cit groups. These data demonstrated that the male offspring of STZ-induced diabetic mothers had low nephron number, tubulointerstitial injury, and renal hypertrophy.

To further understand whether the arginine-ADMA-NO pathway is involved in STZ-induced developmental programming and whether maternal Cit supplementation can prevent it, we examined arginine, citrulline, ADMA, SDMA, and the arginine-to-ADMA ratio in the plasma and kidney of the STZ model rats.

**Figure 4:**
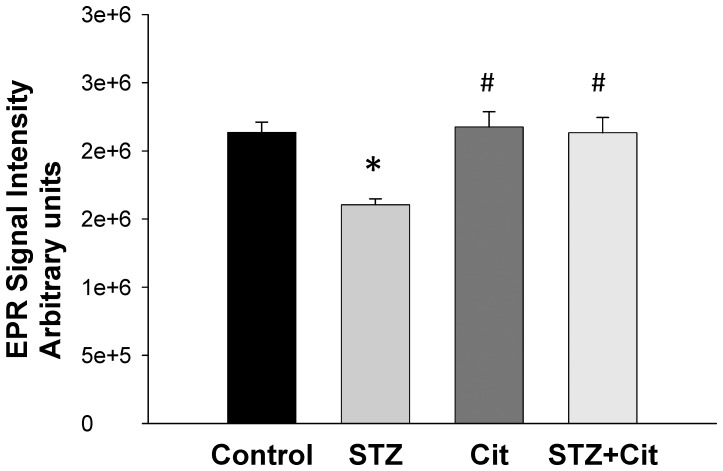
NO production in the STZ offspring kidney detected by electron paramagnetic resonance (EPR). n = 6/group; *p<0.05 vs. control; #p<0.05 vs. STZ.

We found that the plasma levels of arginine, ADMA, and SDMA, and the arginine-to-ADMA ratio were not different among the 4 groups (Table 2). Maternal citrulline supplementation significantly increased the plasma citrulline level in the STZ+Cit group. In the kidney, arginine, citrulline, ADMA, and SDMA concentrations were significantly increased in the STZ group. On the contrary, the renal arginine-to-ADMA ratio was lower in the STZ group than in the controls. Maternal citrulline therapy had no effect on citrulline, arginine, ADMA, and SDMA concentrations in the kidney of controls. However, maternal supplementation with citrulline restored the renal ADMA concentration and the arginine-to-ADMA ratio to normal levels in STZ offspring. As shown in Figure 4, renal NO production detected by EPR was lower in the STZ group than in the controls. Maternal citrulline supplementation significantly increased the NO level in the STZ+Cit group. Similarly, maternal citrulline therapy restored urinary NOx (NO_2_^−^ +NO_3_^−^ ) levels to control levels in the STZ offspring (control: 3.09±0.54, STZ: 0.73±0.22, Cit: 2.54±0.79, STZ+Cit: 3.5±0.23 µM⋅day^−1^⋅100 g^−1^ body weight; control vs. STZ, p<0.05; STZ vs. STZ+Cit, p<0.05). Therefore, maternal citrulline supplementation might protect STZ rats from kidney injury by preserving the ADMA-NO pathway.

**Table 2:** Plasma and renal levels of arginine, citrulline, and dimethylarginine in the offspring of STZ-induced diabetic mothers at 12 weeks of age.

	Control	STZ	Cit	STZ+Cit
	n = 6	n = 6	n = 6	N = 6
Plasma (µM)				
Arginine	90.9±2.6	96.7±3.5	89±3.7	100.8±4.2
Citrulline	66.2±2.6	75.3±7	74.3±3.3	96.2±4.2^a^
ADMA	0.72±0.06	0.78±0.08	1±0.18	1.16±0.17
SDMA	0.51±0.1	0.86±0.11	0.81±0.26	1.33±0.32
Arginine-to-ADMA ratio	129.9±9.5	129±11.5	101.9±15.6	95.6±13.4
Kidney (µM/mg protein)				
Arginine	29.6±3.9	54.5±2.7^a^	28.9±1.9^b^	40.9±2.2^c^
Citrulline	24.4±3.6	37.6±2.7^a^	34.6±4.2	34.1±2.3
ADMA	0.19±0.03	1.29±0.23^a^	0.29±0.12^b^	0.29±0.07^b^
SDMA	0.35±0.03	0.66±0.07^a^	0.52±0.26	0.37±0.05
Arginine-to-ADMA ratio	178.8±32.3	47.3±6^a^	165.9±35.1	231.8±86.9^b^

STZ, offspring of STZ-induced diabetic mother; Cit, offspring of control mother treated with 0.25% l-citrulline; STZ+Cit, offspring of STZ-induced diabetic mother treated with 0.25% l-citrulline.

a p<0.05 vs. control;

b p<0.05 vs. STZ;

c p<0.05 vs. Cit.

**Figure 5:**
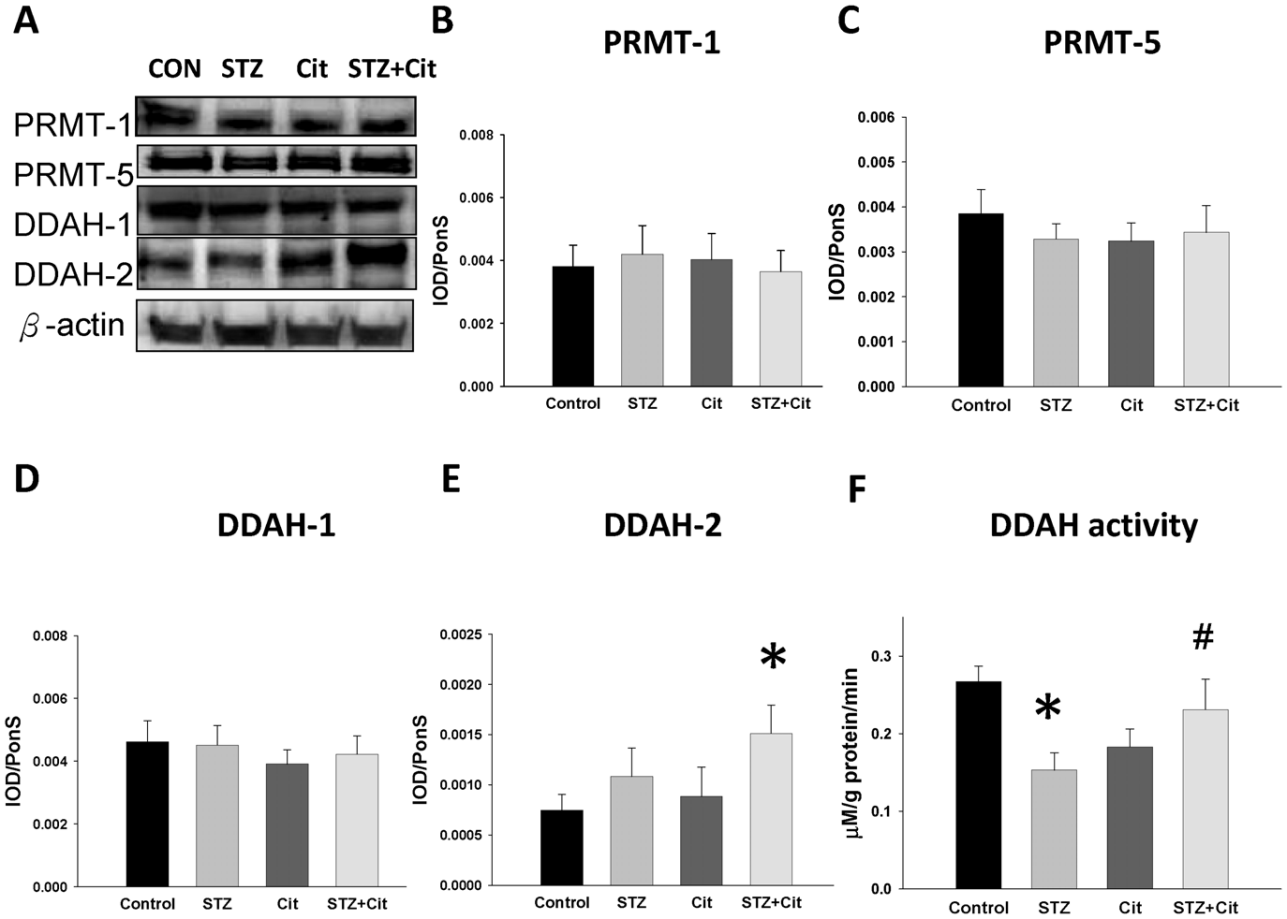
Protein levels and activity of ADMA-related enzymes in the STZ offspring kidney. (A) Representative western blots showing PRMT-1 (∼42 kDa), PRMT-5 (∼72 kDa), DDAH-1 (∼34 kDA), and DDAH-2 (∼30 kDa) bands in control and offspring from mothers treated with STZ and supplemented with l-citrulline at 12 weeks of age. The relative abundances of renal cortical (B) PRMT-1, (C) PRMT-5, (D) DDAH-1, and (E) DDAH-2, and (F) the renal DDAH activity were quantified. n = 6/group; *p<0.05 vs. control; #p<0.05 vs. STZ.

Next, we examined the expression and activity of the proteins involved in the arginine-ADMA-NO pathway. As shown in Figure 5, the protein levels of PRMT-1, PRMT-5 (SDMA-synthesizing enzyme), and DDAH-1 were not different among the 4 groups. However, renal DDAH-2 was higher in the STZ+Cit group than in controls. Moreover, DDAH activity was significantly decreased in the kidney of STZ offspring, which was restored by maternal citrulline supplementation (Fig. 5F). Thus, it is possible that the increase of renal ADMA seen in the STZ offspring may be due to decreased metabolism induced by DDAH.

**Figure 6:**
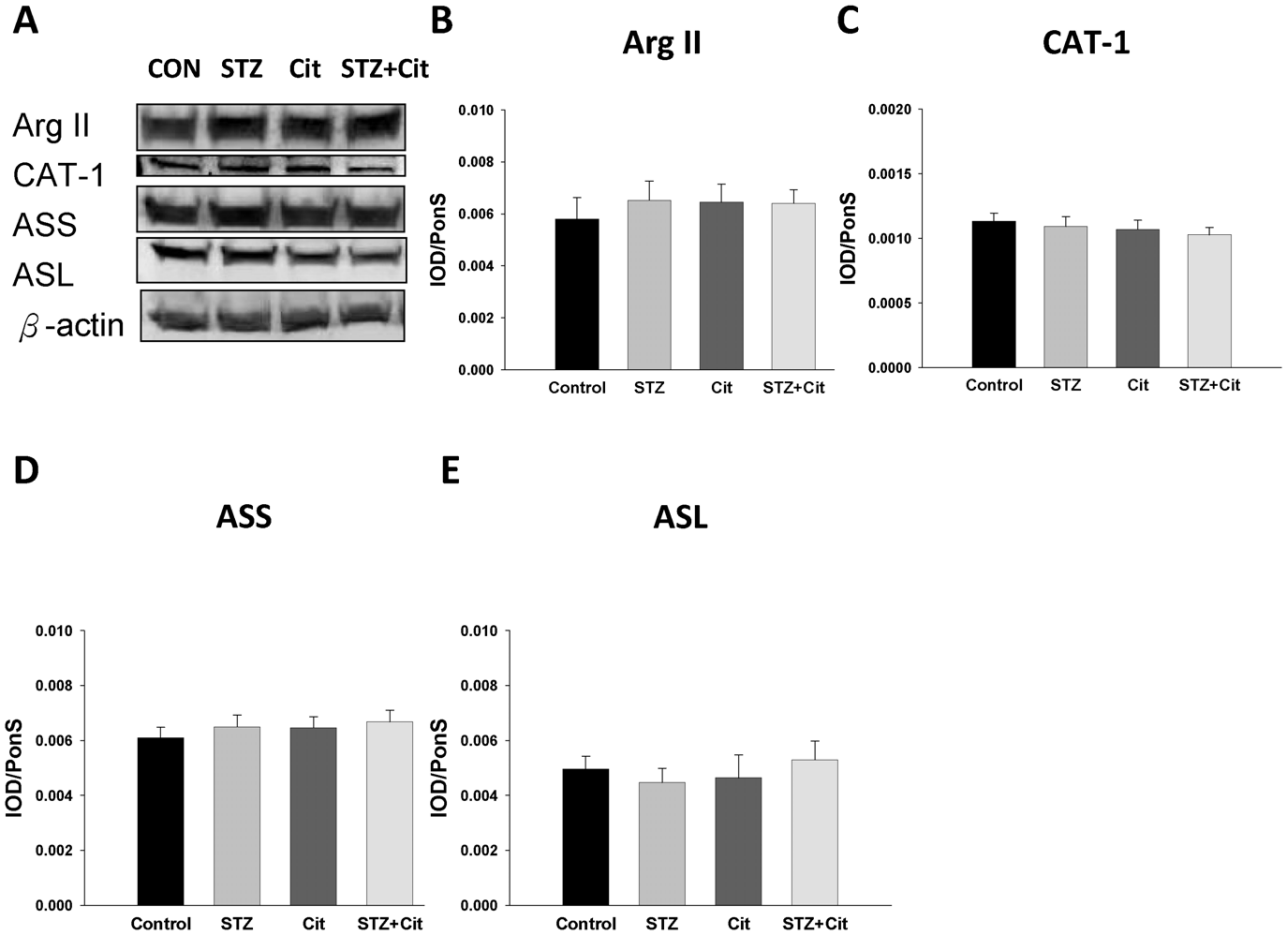
Protein levels of arginine-related enzymes in the STZ offspring kidney. (A) Representative western blots showing arginase II (Arg II, ∼40 kDa), cationic amino acid transporter-1 (CAT-1, ∼68 kDa), argininosuccinate synthase (ASS, ∼50 kDa), and argininosuccinate lysate (ASL, ∼50 kDa) bands. The relative abundances of renal cortical (B) arginase II, (C) CAT-1, (D) ASS, and (E) ASL were quantified. n = 6/group.

Since arginine concentration can be regulated by its synthesis, transport, and consumption, we further investigated whether STZ and Cit therapy can influence arginine-related proteins. As shown in Figure 6, our data revealed that the renal arginase II, cationic amino acid transporter-1 (CAT-1), argininosuccinate synthetase (ASS), and argininosuccinate lyase (ASL) protein levels were not different among the 4 groups.

**Figure 7:**
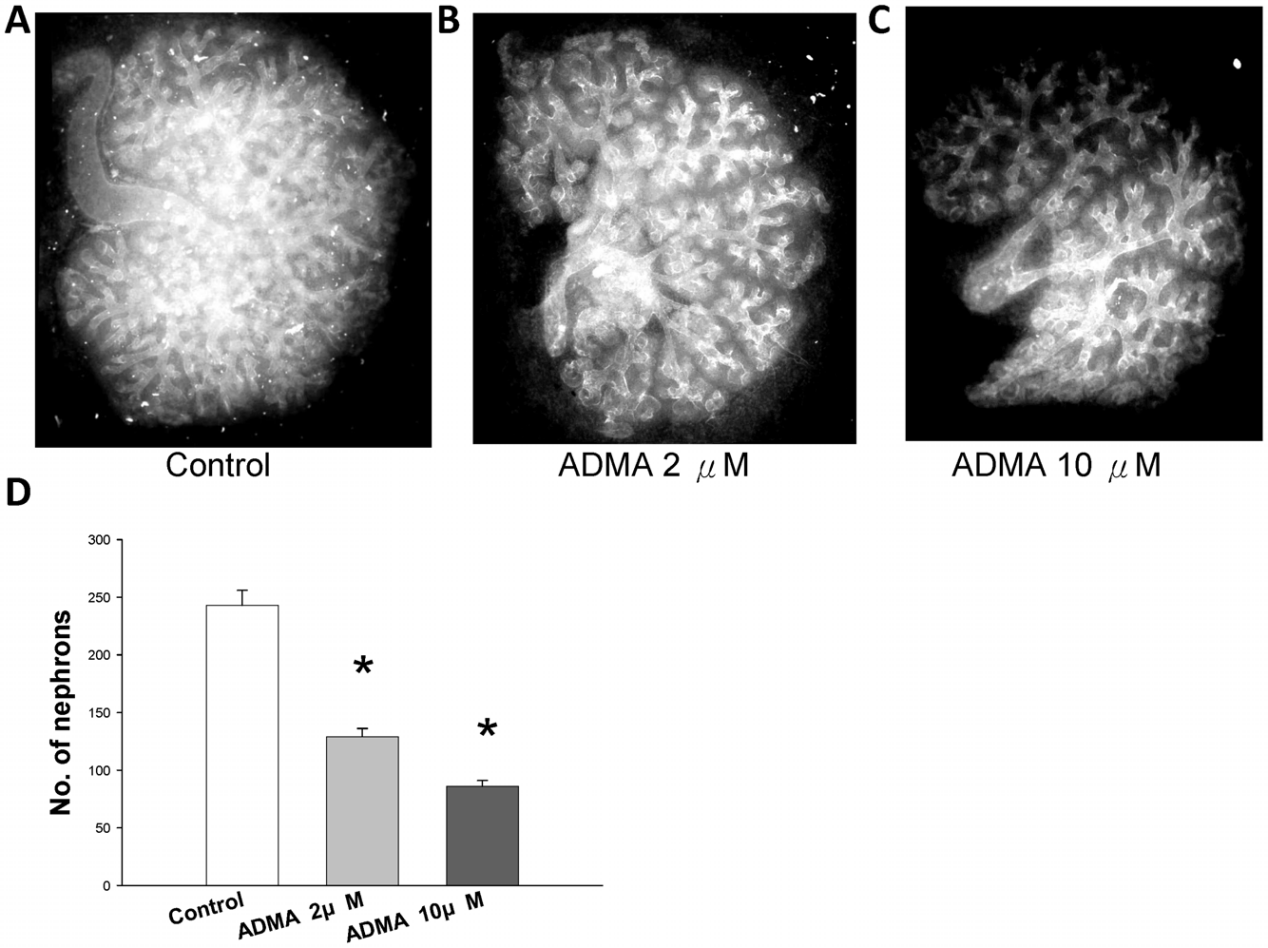
Effect of ADMA on metanephros development. In vitro metanephros development in the (A) control, (B) 2 µM ADMA, or (C) 10 µM ADMA-supplemented medium. (D) The numbers of nephrons were counted. n = 6/group.

We next evaluated whether ADMA can impair nephrogenesis. The morphology of rat metanephroi grown in different concentrations of ADMA is shown in Figure 7. In vitro development was optimal in the standard medium (Fig. 7A). Metanephroi grown in 2 µM ADMA were significantly smaller and contained fewer nephrons (Fig. 7B). At higher ADMA concentrations, fewer nephrons were formed. Moreover, ureteric bud branching morphogenesis was inhibited by ADMA.

## Discussion

**Figure 8:**
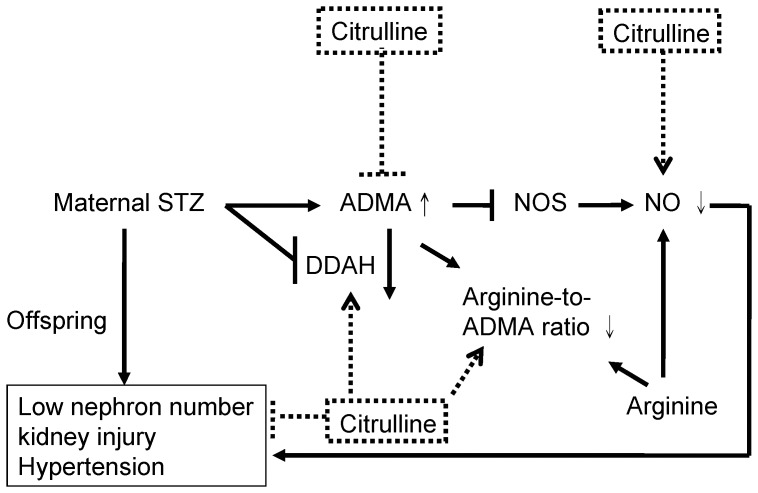
Simplified scheme showing the effects of maternal citrulline supplementation in the STZ offspring kidney. STZ induced an increase of ADMA level, and decreases of arginine-to-ADMA ratio and NO, consequently leading to low nephron number, kidney injury, and hypertension; maternal citrulline therapy decreased ADMA, increased arginine-to-ADMA ratio and NO, and increased DDAH activity in the STZ group to reduce kidney injury and hypertension. Dashed lines indicate the effects of maternal citrulline supplementation on STZ offspring.

The main findings of this study are as follows: (1) the offspring of mothers with STZ-induced diabetes had low nephron number, kidney injury, hypertension, increased ADMA levels, and decreased renal arginine-to-ADMA ratio; (2) ADMA impaired nephrogenesis and reduced nephron numbers in a metanephroi culture; (3) kidney injury, increased ADMA, decreased arginine-to-ADMA ratio, and decreased DDAH activity in the STZ group were reversed by maternal citrulline therapy. Figure 8 is a simple scheme that summarizes our results. It shows that STZ-induced hypertension and kidney injury is related to arginine–ADMA–NO system, which can be restored by maternal citrulline supplementation.

In line with earlier studies, we found that offspring exposed to maternal diabetes during pregnancy exhibited low nephron numbers, kidney injury, renal hypertrophy, and BP elevation [6], [7], [17]. In rat, nephrogenesis occurs predominantly from late gestation to 7–10 days postnatally. Our data showed that 7-day-old nSTZ offspring had low nephron numbers, and ADMA caused a nephron deficit in the kidney of 14-day-old pups. Here, we found for the first time the link between ADMA and low nephron numbers in the STZ-induced diabetic models.

In addition, previous studies demonstrated that the increase of BP and the decrease of renal function slowly progress with age in the majority of the experimental models of developmental programming. Although some studies demonstrated early consequences in offspring exposed to intrauterine diabetes [7], [23], [24], high systolic BP and renal function impairment might not occur until 18 months of age in the offspring of rats with STZ-induced diabetes [17].

Our previous data demonstrated that 50% maternal caloric restriction causes 3 months old offspring develops mild renal dysfunction and a decline in nephron number but not hypertension [8]. These changes are associated with increased plasma ADMA level and decreased arginine-to-ADMA ratio. In the present study, the STZ offspring, at 3 months of age, develops hypertension, renal hypertrophy, and tubulointerstitial injury, but not renal function impairment. Unlike caloric restriction model, increased ADMA and decreased arginine-to-ADMA ratio are prominent in the kidney but not plasma. This discrepancy could be a result of the differential programming mechanism with different models: undernutrition caloric restriction model vs. overnutrition STZ model. However, ADMA accumulation is apparent a common pathway. According to Brenner and Chertow [2], a compensatory hypertrophy occurs in response to nephron deficit to maintain sufficient renal function, leading to hypertension and CKD later in life. Taken together, it is possible that ADMA accumulation is related to low nephron endowment, with the subsequent development of aggressive kidney disease and hypertension, in the setting of in utero exposure to maternal diabetes.

Our study also demonstrated that renal ADMA was increased but plasma ADMA was unchanged in the offspring of STZ-induced diabetic mothers. Despite no change in the protein levels of PRMT-1, DDAH-1, and DDAH-2, the renal DDAH activity was decreased, leading to an elevation of ADMA in the kidney. Importantly, the increase in ADMA can occur early (at age 7 days) in the kidney of nSTZ offspring, during the nephrogenic period, preceding the development of kidney disease and hypertension.

However, there is a discrepancy between renal arginine level and its related protein levels. As shown in table 2, renal arginine level is increased in STZ vs. control. Arginine level in the STZ kidney can be increased because of reduced consumption via other metabolic pathways, increased de novo synthesis, and increased transport. We found no difference of these protein levels related to arginine pathway. However, increased arginine uptake might be due to increased CAT activity instead of increased protein level. Given that the complexity of arginine metabolism, whether increased protein degradation or other metabolic pathways consuming arginine (e.g., *arginine* decarboxylase) are regulated awaits further evaluation.

Given that STZ induces type 1 diabetes while nSTZ causes type 2 diabetes [18], our data indicated that ADMA accumulation in the kidney is a common mechanism involved in in utero exposure to maternal diabetes. Since ADMA treatment can induce glomerular fibrosis contributing to chronic kidney disease progression and hypertension in adult rats [25], our findings further suggest that diabetes induced renal ADMA accumulation in early stage of development might cause the kidney injury and hypertension in later life. The kidney is a major organ that metabolizes ADMA, through DDAH-1 and -2, to maintain a steady level of plasma ADMA [26]. This suggests that ADMA in the kidney correlates better with the consequences of in utero exposure to maternal diabetes than ADMA in the plasma. This may explain why both increased and decreased plasma ADMA levels have been reported in diabetic patients [11]–[14], [27].

Although both PRMT and DDAH have been reported to have a relation with diabetes and its complications [28 29], our results suggest that DDAH activity may have an important role in the development of kidney disease and hypertension in diabetic offspring. A previous report showed that hyperglycemia inhibited DDAH activity to induce ADMA in cultured endothelial cells [30]. Another study also showed that hyperglycemia can cause low nephron endorsement in vivo and in vitro [6]. Given that overexpression of DDAH has an ADMA-lowering effect and improves diabetes-related endothelial dysfunction [31], our data further suggest that restoration of DDAH activity may be a therapeutic target in diabetes-associated programming.

As ADMA and arginine compete for NOS, the arginine-to-ADMA ratio has been used to represent NO bioavailability [32]. We found that renal arginine-to-ADMA ratio, renal NO production (represented by EPR), and the urinary NOx levels were correspondingly decreased in STZ-exposed offspring, suggesting a deficiency of NO in the STZ-induced programming of kidney disease. The administration of arginine is the most common treatment to improve the NO pathway; however, data from human trials are not promising. Given that citrulline can be converted to arginine in the body and that oral citrulline administration can bypass the liver, citrulline supplementation can thus be used as an alternative to augment NO bioavailability [33].

Our other new finding was that elevation of BP, tubulointerstitial injury, increased ADMA, decreased arginine-to-ADMA ratio, and decreased renal DDAH activity in the STZ group were reversed by maternal supplementation with citrulline. On the basis of our observations and those of others, 3 important mechanisms may be involved in the protective effects of maternal citrulline on the developmental programming of kidney disease and hypertension in the STZ offspring: upregulation of DDAH-2 and DDAH activity, reduction of renal ADMA, and restoration of NO bioavailability in the kidney (Fig. 8). Maternal citrulline therapy significantly increased DDAH-2 and DDAH activity and prevented increased ADMA in the kidney of STZ offspring. Given that DDAHs are highly oxidation-sensitive enzymes that can be inhibited by oxidative stress [21], [34] and that our previous observation showed that antioxidants can increase DDAH-2 abundance and DDAH activity to prevent the increase of ADMA in a young bile-duct ligation model [35], our present study suggests that citrulline can restore the disturbed NO/reactive oxygen species balance to augment the DDAH-2 protein level and activity. Consequently, the increase of ADMA in the kidney is prevented by citrulline supplementation. It has been proposed that the blood ADMA level is mainly regulated by DDAH-1, whereas NO is regulated primarily by DDAH-2 [36]. We extend these results to the developmental programming of hypertension and kidney disease and provide the first evidence that citrulline can induce DDAH-2 to increase NO production in the kidney, represented by increased renal arginine-to-ADMA ratio and NO level, in the setting of in utero exposure to maternal diabetes.

In conclusion, our results suggest that maternal diabetes might lead to low nephron numbers in the offspring, which subsequently programs the development of hypertension and kidney disease in adult life. The underlying mechanisms are likely mediated by decreased DDAH activity, increased ADMA, and decreased NO in the kidney. Maternal citrulline therapy might restore the ADMA-NO balance in the offspring, thus preventing the development of hypertension and kidney disease later in life.

## Supporting Information

Table S1 **Antibodies used for Western blotting.**
(DOC)
