# Supplementary material for: Asymmetric Dimethylarginine Is Associated with Developmental Programming of Adult Kidney Disease and Hypertension in Offspring of Streptozotocin-Treated Mothers
Source: PLoS One. 2013 Feb 7;8(2):e55420. doi: 10.1371/journal.pone.0055420 (PMC3567076; doi:10.1371/journal.pone.0055420)
Supplement: Table S1 — Antibodies used for Western blotting. (DOC) [file pone.0055420.s001.doc]

Table S1. Antibodies used for Western blotting.

| Antibody | Host | Source | Dilution |
| --- | --- | --- | --- |
| nNOS | Mouse | Santa Cruz, Santa Cruz, CA | 1:200 |
| eNOS | Mouse | Transduction Laboratories | 1:250 |
| PRMT-1 | Rabbit | Millipore, Billerica, MA | 1:2,000 |
| PRMT-5 | Rabbit | Millipore | 1:2,500 |
| DDAH-1 | Goat | Santa Cruz | 1:500 |
| DDAH-2 | Goat | Santa Cruz | 1:100 |
| Arginase II | Rabbit | Santa Cruz | 1:1,000 |
| ASS | Goat | Santa Cruz | 1:200 |
| ASL | Rabbit | Santa Cruz | 1:200 |
| CAT-1 | Rabbit | Abcam, Cambridge, MA | 1:250 |

nNOS= neuronal nitric oxide synthase, eNOS= endothelial nitric oxide synthase, PRMT-1= protein arginine methyltransferase-1, PRMT-5= protein arginine methyltransferase-5, DDAH-1= dimethylarginine dimethyaminohydrolase-1, DDAH-2= dimethylarginine dimethyaminohydrolase-2, ASS= argininosuccinate synthetase, ASL= argininosuccinate lyase, CAT-1= cationic amino acid transporter-1
